# A Systematic Literature Review of Variables Associated with the Occurrence of African Swine Fever

**DOI:** 10.3390/v17020192

**Published:** 2025-01-30

**Authors:** Sofie Dhollander, Eleonora Chinchio, Stefania Tampach, Lina Mur, Estelle Méroc, Hans-Hermann Thulke, José Abrahantes Cortiñas, Anette E. Boklund, Karl Stahl, Jan Arend Stegeman

**Affiliations:** 1 Assess Department, European Food Safety Authority, 43126 Parma, Italy; 2 P95 Clinical and Epidemiology Services, 3000 Leuven, Belgium; 3 Department of Ecological Modelling, Helmholtz Centre for Environmental Research, 04318 Leipzig, Germany; 4 Department of Veterinary and Animal Sciences, University of Copenhagen, 1870 Copenhagen, Denmark; 5 Department of Epidemiology, Surveillance and Risk Assessment, Swedish Veterinary Agency, 751 89 Uppsala, Sweden; 6 Department of Farm Animal Health, Utrecht University, 3584 CS Utrecht, The Netherlands

**Keywords:** ASF occurrence, explanatory variables, systematic literature review, pigs, wild boar

## Abstract

Since African swine fever virus (ASFV) genotype II reached Europe in 2007 and has widely spread, causing important economic losses to the pig production sector. To guide policy and management actions, robust quantitative evidence about possible explanatory variables associated with ASF in domestic pigs and Eurasian wild boar (*Sus scrofa*) is needed. To this aim, a systematic literature review of the scientific evidence available on variables analysed through quantitative methods investigating their possible association with ASF occurrence was carried out in 2021 and updated in 2024. Information on article metadata, study settings, and details of the analysed variables were extracted from the identified articles. The variables were structured in categories and subcategories, and their frequencies were evaluated, as well as the proportions of the studied variables that proved significant in each subcategory. The literature search retrieved 569 articles, resulting in 48 inclusions in the review after application of the selection criteria. The categories of variables most often significantly associated with the occurrence of ASF in domestic pigs were related to the ASF virus infection pressure in the area, socio-economic factors (mainly human population density and poverty), the pig farming system (pig or farm density and certain biosecurity practises), and wild boar habitats. For wild boars, these were also variables related to ASFV infection pressure in the area, wild boar habitats (mainly climatic conditions, vegetation, waterbodies), and socio-economic factors (especially human population and poverty-related variables). Despite the many studies of variables possibly associated with ASF occurrence, the review identified a gap in quantitative observational studies focusing on manageable variables, i.e., those related to specific biosecurity measures applied to pig farms and during hunting. To allow for a meta-analysis of the results, these studies should be performed according to standardised protocols using harmonised data collections.

## 1. Introduction

Since African swine fever (ASF) virus genotype II reached Georgia in 2007, it has spread extensively across Europe, Asia, and into the Americas (Haiti and the Dominican Republic), leading to considerable economic losses in the pig industry. The World Organisation for Animal Health reported in its summary report of the global ASF situation during the period from January 2022 to 23 August 2024 that ASF had been reported in 61 countries across five continents, causing around 1.7 million animal losses globally, including domestic pigs and wild boars [1] The current lack of effective vaccines and treatments implies that the understanding of the risk factors associated with ASF occurrence, spread, and persistence in both domestic pigs and wild boar populations plays a pivotal role in the prevention and control of the disease. Robust evidence on those explanatory variables associated with ASF occurrence is needed to inform risk mitigation measures and policies.

The extensive spread of ASF worldwide has dramatically increased the number of publications on variables possibly associated with the occurrence of ASF in recent decennia. Recent examples are a narrative review of [2] on potential environmental risk factors for the occurrence of ASF in wild boars; [3], providing a literature review of risk factors for the incursion of ASF in pig farms, also including assessments based on expert opinion; and [4], who combined a modelling framework with records of ASF cases and multiple covariates to predict the risk distribution of ASF at a global scale. Those examples use different assessment methodologies, going from qualitative analytical approaches to quantitative predictive modelling.

The objective of this systematic literature review (SLR) was to provide an overview of the available quantitative studies on variables associated with ASF occurrence in domestic pigs and wild boars based on field observations using information collected both from ASF-affected and ASF-free areas or farms. The results of this review aim to summarise the quantitative evidence currently available on explanatory variables associated with ASF occurrence in domestic pigs and wild boar populations, useful to inform risk mitigation strategies and policies. Furthermore, the outcome highlights the existing knowledge gaps and research questions that deserve further investigation.

## 2. Methods

A systematic literature review was performed to address the following review question: what are the variables associated with the occurrence of ASF in domestic pigs and wild boar populations? The review followed EFSA and PRISMA guidelines for systematic reviews [5,6] and was performed in © 2025 DistillerSR Inc (Evidence Partners) software for systematic literature reviews.

Queries were performed in Web of Science Core Collection (Science Citation Index Expanded, Conference Proceeding Citation Index—Science, and Emerging Sources Citation Index), PubMed, Scopus, and Cab Abstracts databases in 2021 and updated on 29 February 2024.

The search string was created combining keywords following the PECO structure for observational studies, including the population of interest (i.e., wild boars and domestic pigs), generic terms to identify the variables to which these populations have been exposed and that were investigated in the studies (or not exposed and compared with), and the outcome of exposure (ASF occurrence/reoccurrence/persistence/spread). These elements were joined with “AND” statements, and the search strings were adapted for each database to narrow results as much as possible while maximising the number of relevant returned studies (see Supplementary Materials Table S1 for the full electronic search strategy).

Eligibility criteria for inclusion in the review were a priori defined as follows: (i) primary peer-reviewed research studies; (ii) studies based on quantitative methodologies applied to field data (i.e., quantitative data collected of the potential variables associated with ASF occurrence and quantitative analytical techniques applied to obtain measures of association for ASF in pigs or wild boars); and (iii) studies published in English. No limitations concerning the year of publication were applied.

**Figure 1:**
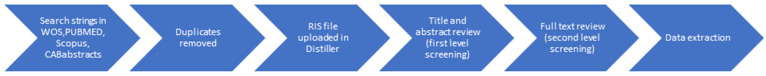
Overview of the systematic literature review process.

Studies not relevant to the research question (e.g., focusing on animal species other than domestic pigs and wild boars or not related to variables associated with ASF), not providing quantitative data on ASF-associated variables, not providing original data (e.g., reviews, editorials, letters), lacking a detailed description of the study design and/or the results of the analyses, and studies for which the full text was not available online were excluded. The study selection process is summarised in Figure 1.

Duplicated articles retrieved from multiple databases were removed using EndNote 21.4.0/software. Articles were evaluated for eligibility in accordance with the inclusion/exclusion criteria described above. The first level of screening involved the independent screening of titles and abstracts by two of the authors (i.e., two reviewers per study). Publications judged to be relevant by only one reviewer were discussed among reviewers until a consensus was reached about their selection for further screening. Publications rejected by both reviewers were excluded. Whenever the title or abstract did not give a clear indication of relevance, the full text was screened. The second level of screening involved the screening of full text articles identified in level 1, with one reviewer per study. Both level 1 and level 2 screenings involved an initial phase of harmonisation and training regarding the assessment of study eligibility criteria, across all screeners of each objective.

Data were extracted from relevant papers using a standardised form in Distiller^®^. One, where a reviewer per study individually extracted data and metadata, including the following:

- Study setting, i.e., country, region, study period;
- Study characteristics, i.e., study design, target population (wild boars or domestic pigs), epidemiological unit of interest (farm, individual animal, or population), study sample size, and variables investigated;
- Statistical analysis, i.e., type of statistical analysis (e.g., logistic regression model, generalised linear mixed model), and measure of association of the outcome (e.g., OR, RR, HR);
- Study results, including CIs and *p*-values.

All variables were then categorised in macro-categories and subcategories to facilitate their analysis.

To be able to summarise the results of the literature review, we focused the significance of variables possibly associated with ASF occurrence that were evaluated by a given model. Therefore, the variables were first grouped in 7 main categories (ASFV infection pressure, timing of the study, pig farming system, socio-economic factors, wild boar management, wild boar habitat, and arthropods) and two levels of subcategories. For each category and subcategory of the variables, frequency indicators were calculated, including the number of studies where a variable was analysed, the number of studies where this variable was significantly associated with the occurrence of ASF, and the proportion of studies where this variable was significant over the number of studies where it was investigated.

The SLR did not include an analysis of the quality of studies in terms of methods (risk of bias) and reporting.

## 3. Results

**Figure 2:**
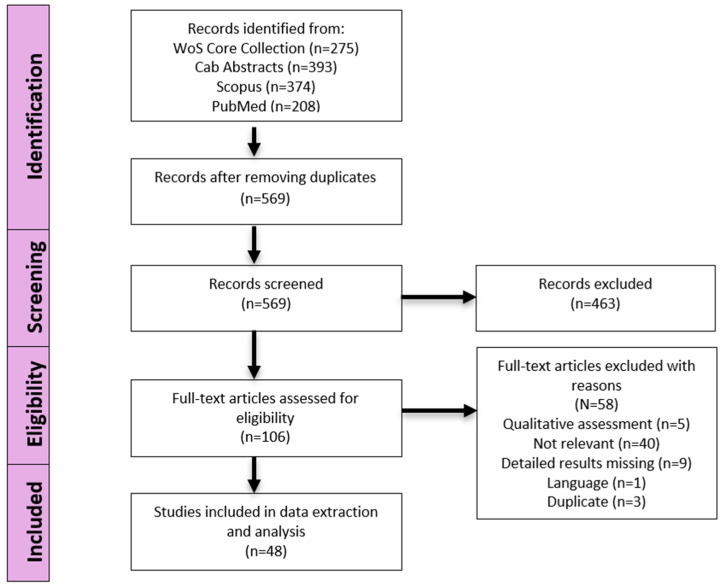
Literature search flow diagram showing the selection process of eligible articles on quantitative studies of variables possibly associated with ASF occurrence. WoS = Web of Science.

The literature search retrieved 569 articles related to putative quantitative variables associated with ASF occurrence in domestic and/or wild boar populations. The study selection process was carried out according to the PRISMA statement (Moher et al., 2009) and is reported in the flow chart shown in Figure 2.

From the 569 articles, only 48 articles were included in the analyses, from which data were extracted in a standardised form. Of these, 34 (45 studies) described variables possibly associated with ASF occurrence in domestic pigs, 17 articles (41 studies) described variables possibly associated with ASF occurrence in wild boars, and three articles (four studies) described variables possibly associated with ASF occurrence in both populations.

Half of the selected articles (*n* = 24) described studies conducted in Europe, with some of them describing studies in more than one country. Countries that were most covered in those studies included Estonia (*n* = 8), Italy (*n* = 5), Latvia (*n* = 3), Lithuania (*n* = 4), Poland (*n* = 4), and Romania (*n* = 5). Sixteen studies focused on the situation in Asian countries (China (*n* = 3), Indonesia (*n* = 3), Malaysia (*n* = 1), South Korea (*n* = 3), Russia (*n* = 4), and Vietnam (*n* = 2)). Eight articles studied variables in African countries and two articles had a global perspective.

The 48 relevant studies used 19 different statistical models to study the variables possibly associated with ASF occurrence in the pig or wild boar populations (Supplementary Materials Table S2). In some articles, more than one statistical model was used. It was decided to keep the habitat niche models included [7,8,9,10], although these models retain only variables with the highest explanatory power for ASF occurrence. This does not necessarily classify them as risk factors. Consequently, in this review, the term “risk factor” was deliberately avoided.

There were many different studies analysing variables possibly associated with ASF occurrence in pigs and wild boars (697 variables–study combinations). However, there were very few studies investigating the same variables with the same study design in the same population. To illustrate this, there were for instance eight studies that studied variables related to “Access to the pig farms”, of which five found significant relations. However, the investigated variables were different (i.e., gate at entry of farm (*n* = 2); lock for each pig pen (*n* = 1); premises not fenced (*n* = 4); wild birds accessing pig pen (1)). Unfortunately, several studies did not provide a quantitative estimate of the variables’ association with ASF occurrence. Or, the quantities of association were different, e.g., risk or predictor; hence, a meta-analysis was not performed.

### 3.1. Domestic Pigs

**Figure 3:**
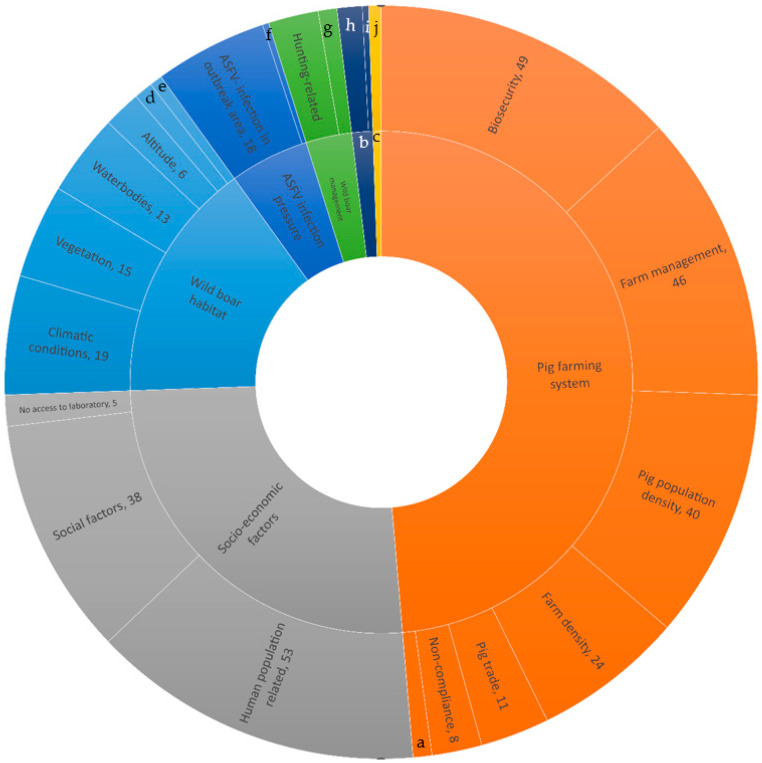
Number of studies studying variables possibly associated with the occurrence of ASF in domestic pigs. a = pig characteristics; b = timing of the study; c = arthropods; d = wild boar suitability; e = fauna; f = abattoir in the commune; g = wild boar abundance; h = outbreak phase; i = sampling period; j = ticks.

We identified seven categories of variables that were grouped into 23 subcategories and 62 second-level subcategories. The absolute numbers of variables per category and first subcategory are presented in Figure 3. Globally, three categories have been studied more frequently, namely those related to the pig farming system (*n* = 181), socio-economic factors (*n* = 96), and those related to the wild boar habitat (*n* = 58). To a lesser extent, this was followed by categories related to wild boar management (*n* = 11), the timing of the study (*n* = 5), and arthropods (*n* = 2). Figure 3 also shows the absolute numbers of variables studied per the first subcategory.

Per category, the number of significant variables over those studied were (i) the pig farming system (98 significant variables/181 studied variables), (ii) socio-economic factors (64/96), (iii) the wild boar habitat (43/58), (iv) ASFV infection pressure in the area (13/19), (v) wild boar management (3/11), (vi) the timing of the study (5/5), and (vii) arthropods (2/2). 

**Figure 4:**
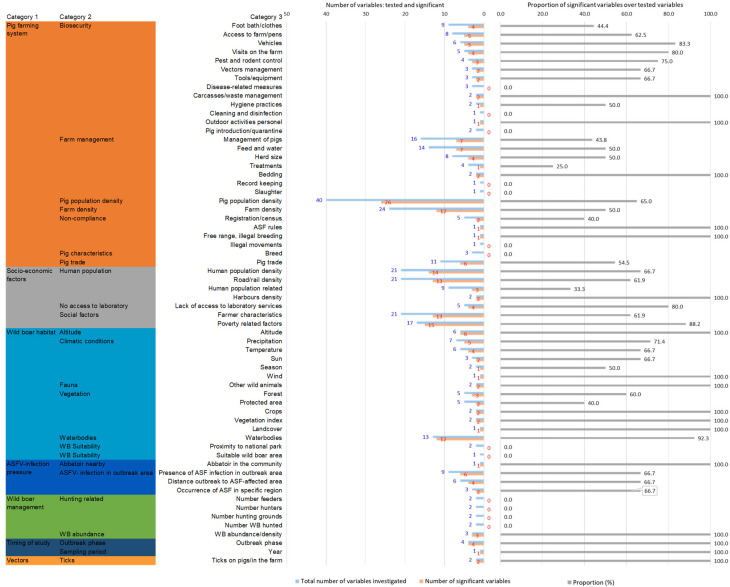
Number of significant variables and number of variables studied per category and subcategories 1 and 2 for ASF occurrence in domestic pigs.

Figure 4 displays the number of variables that were studied and found to be significant for subcategory 2. In the category of the pig farming system, the two subcategories with the most significant variables associated with the occurrence of ASF were the pig population density (*n* = 26) and farm density (*n* = 12). Clearly, these were the most frequently studied variables as well, with 40 and 20 studies each, respectively. Pig densities in different farming/production types were found to be significant (e.g., indoor farms, outdoor farms, semi-extensive farms, or free-ranging farms; see Table 1).

Biosecurity is a very broad subcategory including many different variables that could potentially be associated with ASF occurrence. The most often significant variables were the consistent application of foot bath/changing cloths (*n* = 9, sign. =4), access to the farm or pens (*n* = 8; sign. = 5), vehicles entering the farm (*n* = 6; sign. =5), and other less studied variables, which can be seen in Figure 4 and Table 1 for more details.

Other variables found to be significant in the context of pig farming were farm management (*n* = 46, sign. = 21) (e.g., management of pigs, type of feed and water provided to the pigs, herd size, and housing system), non-compliance with sanitary rules (*n* = 8; sign. = 4), and pig trade (*n* = 11; sign. = 6) (Table 1 for details).

In the category of socio-economic factors, social factors were frequently found to be significantly associated with ASF occurrence (*n* = 38, sign. = 28), followed by human population (*n* = 53, sign. = 32) and no access to laboratory services (*n* = 5; sign. = 4). The subcategory of social factors included variables related to the farmer characteristics (*n* = 21, sign. = 13), such as lower age and education level, as well as poverty-related factors (*n* = 17; sign. = 15), like the deprived conditions of a region or unemployment rates.

Further categories studied for domestic pigs were the wild boar habitat (*n* = 58, sign. = 43), i.e., vegetation, water bodies, fauna, and climatic conditions, ASF infection pressure in the area, including ASFV infection in the area or the presence of abattoirs (*n* = 19; sign. = 13), wild boar management (*n* = 11; sign. = 3), the presence of arthropods (*n* = 2; sign. = 2), and the timing of the study, i.e., studying the impact of the outbreak phase or the year of the study on the occurrence of ASF in pigs (*n* = 5, sign. = 5) (Figure 4).

Further details about the individual significant variables in subcategory 3 and the direction of their effect on ASF occurrence in the domestic pig holdings are summarised in Table 1.

Where needed, the continuous significant variables were reformulated so they all had a positive effect on the occurrence of ASF in pig farms to make the interpretation easier. For instance, “Decreasing distance to outbreaks in DP (2)” should be interpreted as follows: a decreasing distance to other outbreaks in domestic pigs (DP) had a significant (positive) effect on the occurrence of ASF on the pig farms in two studies. Similarly, “Increasing herd size (2)” indicates that larger pig farms had a significant (positive) effect on the occurrence ASF in pig farms in two studies.

The binomial variables (e.g., presence or absence) were formulated to indicate a positive effect on the occurrence of ASF. For instance, in Table 1, “Swill feeding (3)” should be interpreted as the implementation of swill feeding on pig farms having a significant (positive) effect on the occurrence of ASF in three studies.

Important results are 50 significant variables that can be managed by the farmers in the biosecurity and farm management categories all positively associated with ASF on the farms.

### 3.2. Wild Boars

**Figure 5:**
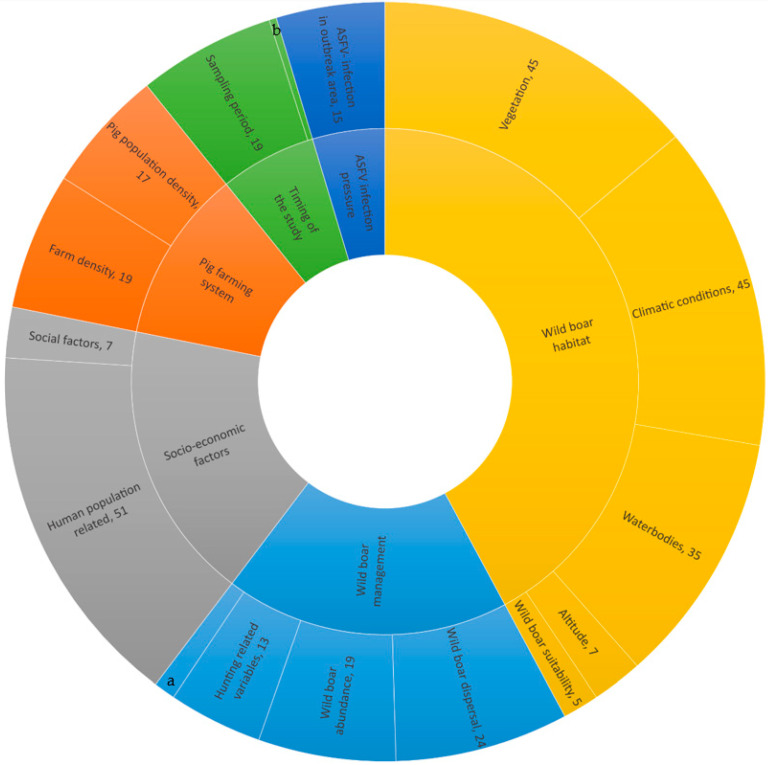
Number of studies investigating variables possibly associated with ASF occurrence in wild boars. a = wild boar population characteristics; b = outbreak phase.

**Figure 6:**
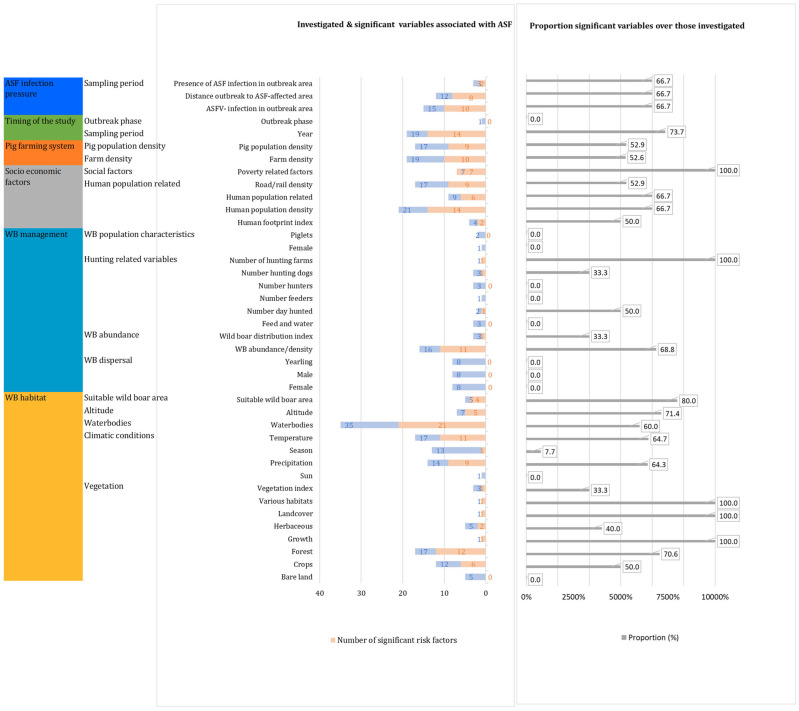
Number of significant variables and number of variables studied per category and subcategories 1 and 2 for ASF occurrence in wild boars.

We identified six categories of variables for wild boars, which were grouped into 16 subcategories and 40 second-level subcategories. For wild boars, the absolute number of variables studied per categories and first subcategories are presented in Figure 5. The most studied categories were the wild boar habitat (*n* = 137), wild boar management (*n* = 59), socio-economic factors (*n* = 58), and variables related to the pig farming system (*n* = 36). Less studied were the timing of the study (*n*= 20) and the ASFV infection pressure in the area (*n* = 15). The absolute numbers of variables studied in the different subcategories are shown in Figure 5. Figure 6 shows the proportions of significant variables over those that were studied for ASF in wild boars per category and subcategories 1 and 2.

Per category, the number of significant variables over those studied were (i) the wild boar habitat (75 significant variables/137 studied variables), (ii) wild boar management (15/59), (iii) socio-economic variables (38/58), (iv) the pig farming system (19/36), (v) the timing of the study (14/20), and (vii) ASF infection pressure (10/15).

In the category of the wild boar habitat, most studied variables related to the vegetation (*n* = 45; sign. = 24), climatic conditions (*n* = 45; sign. = 21), water bodies (*n* = 35, sign. = 21), and wild boar suitability areas (*n* = 5; sign. = 4).

For the category of wild boar management, only 15 out of 59 investigated variables were significant, and most of these were related to wild boar abundance (mainly based on the number of hunted wild boars) (*n* = 16, sign. = 11).

In the category of socio-economic factors, human population-related variables (*n* = 51, sign. = 31) were most studied, and social factors related to poverty (*n* = 7, sign. = 7) were found to be all significantly associated with ASF in wild boars.

In the category related to pig farming, all the significant variables for the occurrence of ASF in wild boars were in the subcategories of farm density (*n* = 19, sign. = 10) and pig population density (*n* = 17, sign. = 9).

Table 2 provides a summary of the individual significant variables for ASF occurrence in wild boars per subcategory 3, with the references of the studies where these were investigated. Further details about the direction of their effect on ASF occurrence in wild boars can be found in the table.

## 4. Discussion

Forty-eight articles were found to be eligible for this study, which is surprisingly low considering the economic importance of the disease. All these publications were published between 2011 and 2024, with half of them in the years 2020 to 2022. This indicates that good studies investigating variables quantitatively associated with ASF occurrence are difficult and resource-intensive. Twice as many papers studied variables possibly associated with the occurrence of ASF in domestic pigs (*n* = 34) than in wild boars (*n* = 17). This could be associated with the easier collection of harmonised data from pigs than from wild boar populations. In addition, ASF in wild boars has only been considered a relevant topic during the last 10–15 years because previously, it was not considered that ASF would become endemic in wild boar populations.

The 48 eligible studies found a substantial number of variables significantly associated with the occurrence of ASF in pigs (228 variables) and wild boars (171 variables). The overall proportion of variables that were significant over those that were studied was higher in pigs than in wild boars (prop = 0.6 in pigs in versus 0.5 in wild boars). This reflects a better understanding of factors driving ASF in domestic pigs compared to wild boars.

We did not perform a meta-analysis due to the heterogeneity of the variables considered across the studies. This heterogeneity encompassed the type of the assessed variables (e.g., climatic conditions, human population-related variables, or vegetation indexes), definitions used across studies, study design, and temporal and spatial resolution. Particularly challenging was the category of biosecurity, where inconsistent definitions of farm types, practises, and categories across studies hindered meaningful comparisons. This underpins the call for the use of standardised study designs, ontologies, and data collection methods for epidemiological investigations of ASF outbreak farms or wild boar habitat areas.

In addition, we chose not to assign weights to the variables due to subjective criteria required to establish such weights. For example, should weighting be based on the sample size or the number of studies? This may introduce bias, as some case–control studies, although expensive and limited in size, provide more valuable insights into risk factors compared to larger but less specific studies such as random forest analyses.

Instead, we adopted a descriptive approach to summarise our findings. While this approach facilitated the presentation of results, it is important to acknowledge its limitations. Firstly, the restriction of the inclusion of only studies with the title and abstract published in English could have led to some bias, as some studies may have been published in different languages that could have had valuable results on possible variables associated with ASF on commercial pig farms.

Secondly, a variable may not reach statistical significance, e.g., due to study power or design, while still having a genuine effect. To provide a more nuanced understanding of the evidence, a comprehensive evaluation of the impact of a variable on a specific outcome should consider not only statistical significance but also effect sizes and confidence intervals [50]. These metrics address the magnitude of the effect and the associated uncertainty.

Thirdly, several studies lack formal quality assurance, e.g., excluding factor collinearity or an evaluation of risk of bias. Together with restricted access to the underlying datasets, this limits our ability to appraise the strength of the evidence via meta-analysis.

### 4.1. Discussion-Domestic Pigs

Some categories addressing the occurrence of ASF in domestic pigs were intrinsically related to the epidemic itself. One category was the timing of the study, which covers variables associated with the subcategories of outbreak phase or sampling period. These subcategories were infrequently studied and indeed, it can be argued that they include mere descriptors of the epidemic. Not surprisingly, the category had the highest proportion of significant variables; indeed, outbreaks are more likely to occur during the epidemic’s expansion compared to later phases with an established perpetuation of infections.

The second category of ASF infection pressure in the area also includes a high proportion of significant factors, most of them related to the subcategory of ASFV infection in the outbreak area. Non-surprisingly, the ASFV infection pressure in the area is an important risk factor for ASF outbreaks in pigs.

The variables primarily investigated in pig populations were human-driven, i.e., the socio-economic factors category and the category of the pig farming system. Farmer characteristics (e.g., education levels or relationships with other farmers) and poverty-related descriptors of the outbreak area (e.g., the low material deprivation index, unemployment rate, or micro-criminality in the area) were frequently investigated and often significant. This illustrates that different social backgrounds influence the risk of ASF outbreaks in pigs. However, it must be considered that many of the poverty-related variables were analysed in the same study area, i.e., Sardinia [20; 15], which is characterised by specific swine production types and a long history of ASF. Therefore, further research is needed to verify the importance of societal variables in other regions. Human population density was frequently investigated and often found to be a significant variable for the occurrence of ASF in pigs, further emphasising the role of human activities in disease dynamics. Human population density has been analysed using both direct measures and proxies such as the presence of roads, urban areas, and human footprint indices. One study in Sardinia [15] identified human population density as a protective factor. Cappai et al. argue that illegal pig breeding may be more prevalent in less populated mountainous areas, likely due to the remoteness and reduced oversight in such regions. It is crucial to consider the causal relationship between socio-economic variables and disease risk. Is poverty a direct driver of risk or does it influence disease risk indirectly through its impact on production systems? This highlights the importance of tailoring disease control measures to specific socio-economic contexts. Participatory approaches may offer numerous advantages for this purpose, as they allow one to identify specific ASF prevention measures that are practical, feasible, and acceptable by local farmers in diverse regions.

In the category of the pig farming system, the most studied subcategories were biosecurity, pig population, farm density, and farm management. The highest proportions of significant variables were found for descriptors of increasing pig and farm density. In the biosecurity subcategory, some variables showed high proportions of significant study outcomes. Examples are carcass/waste management and outdoor activities of the personnel (proportions both one). Other subcategories had more moderate proportions, such as controlled access to the farms (proportion = 0.6), vehicles entering the farm (0.5), the consistent use of foot bats and changing clothes, and the number of visitors (both proportions 0.4). Similarly, moderate significant proportions in the farm management subcategory of feed and water (proportion = 0.5) and the management of pigs (proportion = 0.4) were found. These subcategories are all known examples of good biosecurity practises and therefore, intuitively higher proportions would be expected. There were only a few controlled studies evaluating the effect of implementing biosecurity farming practises on the risk of ASF occurrence on the farms. These studies are costly, and it is difficult to obtain a sufficiently large sample size. Unfortunately, unlike most other subcategories, these subcategories include variables that are manageable by the farmers. Some practises can have a protective effect (e.g., controlled access to farm and pens, the routine cleaning of the pig pen, and vector control), and others are a potential risk factor (e.g., inadequate carcass management, swill feeding, and no separation of sick pigs).

The category of the wild boar habitat contains a relatively high proportion (0.7) of significant variables for the occurrence of ASF in domestic pigs, with 43 out of the 58 studied variables being significant. However, whether that relates directly to the presence of ASF in domestic pigs or indirectly through the potentially affected wild boar population is unclear.

While certain types of factors for the occurrence of ASF in pigs have been extensively studied, others need further investigation, such as those related to the role of arthropods as potential vectors, for which very few quantitative studies exist.

### 4.2. Discussion-Wild Boars

As for domestic pigs, the categories “Timing of the study” and “ASF infection pressure” returned very high proportions of significant variables among those studied. As already discussed above, this is due to the inherent link between the epidemic phase and outbreak incidence, as well as the significant role of infection pressure in driving outbreaks in both pigs and wild boars. The categories most studied as potential variables for the occurrence of ASF in wild boars were the wild boar habitat, wild boar management, socio-economic factors, and, to a lesser extent, farming systems.

Of these, the socio-economic factors category had the highest proportion of significant variables (0.7). Half of the studied variables in the wild boar habitat and pig farming system were significant, with only a smaller proportion of 0.3 in wild boar management. Interestingly, only 15 wild boar management variables out of 59 studied variables were found to be significant for the risk of ASF in wild boars. Most of these (11) were related to wild boar abundance (mainly based on the number of hunted wild boars). This could be partly explained by the difficulty of obtaining good quality standardised data on wild boar populations and management practises of those populations, or it could be related to inadequate study designs. In this sense, habitat and suitability modelling by ENETWILD and other research consortia is essential for standardising data collection methods and the provision of georeferenced wild boar abundance estimates.

Unfortunately, this does not allow us to propose specific management practises for hunters that would reduce the risk for ASF in wild boars, besides keeping wild boar abundance under control to avoid increased wild boar densities.

Bergmann et al. [2] published the outcomes of an SLR on ASF risk factors in wild boars and domestic pigs. Although the objectives of their review were similar, the methodology was quite different. Bergmann et al. extracted data on “potential” and “Observed” risk factors mentioned in the literature both with and without quantitative observations. The outcomes of both types of risk factors were compared. The analysis focused on the total number of reported factors rather than their statistical significance. When comparing the results of the totals of the observational-based risk factors with the absolute numbers of variables extracted from quantitative studies in this review (Figure 5), both SLR’s show the same top categories, namely “Environment” (called wild boar habitat and wild boar management in this study), “Husbandry and biosecurity” (called pig farming in this study), and “Society” (called socio-economic factors in this study). An addition of our study is the documentation of the proportion of significant variables among those studied. Bergmann et al. (2022) mentioned the potential overrepresentation of the environment category in their review and encouraged researchers to investigate new putative risk factors for the occurrence of ASF in wild boar populations. In this SLR, 60% (196/340) of all the studied variables possibly associated with the occurrence of ASF in wild boars were related to the wild boar environment. Among the environmental variables, 13% (26 out of 196) were analysed using niche habitat models [8,9].

### 4.3. Overall Conclusions

This SLR provides a comprehensive overview of the variables investigated in quantitative epidemiological studies and found to be associated with ASF occurrence and spread in both pig and wild boar populations. The results can guide policy and management actions, as well as future research efforts to address knowledge gaps. It clearly demonstrated that certain categories, such as socio-economic factors, the pig farming system, and the wild boar habitat and management, are more extensively studied, while others, such as the role of arthropod vectors, require further investigation.

The subcategories for ASF occurrence in pigs with high proportions of significant variables over the number of variables studied were those related to ASF infection pressure in the area, socio-economic factors (mainly human population density and poverty), the farming system (mainly certain biosecurity measures and farm management practises and pig and farm density), the wild boar habitat (mainly climatic conditions, vegetation, and water bodies), and wild boar abundance.

The take home message for pig farmers is that this review confirms that NOT performing common biosecurity measures, such as implementing physical barriers to farm premises and pig sheds (fence/gate) for people, pets, rodents, wildlife, and arthropods; chemical barriers (washing and disinfection of cloths, footwear, vehicles, tools); appropriate farm and slaughter waste management in closed containers; and avoiding contamination of bedding and feed in storage places or during free ranging, all had a positive association with ASF occurrence in the pig farms.

In addition, farm management practises such as poor disease prevention and control (e.g., no quarantine or culling of sick animals) or all practises that relate to increased movements of people and animals that can be vehicles for ASF to enter the farm (e.g., visits of family and friends or professionals, exchange of boars for mating, staff involved in outdoor activities) increase the risk of ASF occurring on the farms.

The highest vigilance is needed at any point of time. Severe controls are needed to check if these biosecurity measures are properly implemented on commercial farms, particularly in farms located in poorer areas, in areas with high pig or wild boar density or with nearby ongoing ASF outbreaks, or during the warmer period of the year.

For wild boars, the highest proportions were observed in ASF infection pressure in the area, socio-economic factors (human population density and poverty), the wild boar habitat (altitude, crops, forest, wild boar suitability), and, to a lesser extent, wild boar management, with wild boar abundance (mainly based on the number of hunted wild boars)) playing the biggest role.

The review also highlighted the lack of research or good field data on some variables possibly associated with ASF occurrence, such as biosecurity (i.e., specific farm practises) and wild boar management data (specific hunting practises). This is a fundamental gap that needs to be addressed urgently, as these are factors that could be mitigated more readily compared to population- or habitat-related factors.

Although many publications have addressed risk factors for ASF over the past decade, including narrative reviews or qualitative or expert opinion studies, quantitative studies have been less frequent. This may have contributed to the observation that certain factors have been widely regarded as conferring risk, while quantitative evidence is still lacking. In addition, the comparability of the different variables investigated in these studies was low, preventing a meta-analysis to be carried out. However, it is acknowledged that lacking quantitative research on potential risk factors does not mean they do not play a role in the epidemiology of ASF.

Despite the many publications on variables possibly associated with ASF occurrence, observational studies focusing on variables manageable by farmers are highly needed, i.e., those related to specific biosecurity measures applied on pig farms. There are tools available that can support a harmonised collection and analysis of data related to biosecurity measures and African swine fever on commercial pig farms, such as BIOCHECK or SIGMAm, which could facilitate a meta-analysis of studies performed in different settings and epidemiological contexts.

## Figures and Tables

**Table 1:** Variables studied for their possible association with ASF occurrence in domestic pig holdings, analysed in 34 published papers.

Category		Subcategory 2	No. Studies Where Variable Was Investigated	No. Studies Where Variable Was Significant	Proportion *	Significant Variable (No. of Studies Where Specific Risk Factor Was Significant)	References
	Subcategory 1						
Timing of study			5	5	1		
Sampling period		Year	1	1	1.0	2008 versus 2009 (1)	[11,12,13]
Outbreak phase		Outbreak phase	4	4	1.0	Second year of outbreak (1); third year of outbreak (1); infection previous year (2)	
ASF infection pressure in outbreak area			19	13	0.7		
ASFV infection pressure in outbreak area		Abattoir in commune	1	1	1.0	Abattoir in commune (1)	[14,15,16,17,18]
		Distance outbreak to ASF-affected area	6	4	0.7	Decreasing distance to outbreaks in DP (2); decreasing distance to outbreaks in WB (2)	
		Presence of ASF infection in outbreak area	9	6	0.7	ASF in neighbouring areas (3); increasing ASF prevalence in wild boars (2); increasing monthly incidence in wild boars (1)	
		Occurrence of ASF in specific region	3	2	0.7	Occurrence in district Busia in Kenya (1); occurrence in district Busia in Uganda (1)	
Socio-economic factors			96	64	0.7		
Lack of access to laboratory services		Lack of access to laboratory services	5	4	0.8	Increased distance to the nearest diagnostic lab (2); increased time needed for diagnostic test (2)	[15,19]
Social factors		Farmer characteristics	21	13	0.6	Decreasing age of the farmer (3); lower education (5); male (3); increased number of relationships with other farms (2)	[15,20]
		Poverty-related factors	17	15	0.9	Low material deprivation index (8); low employment rate (1); increasing micro-criminality (3); increasing number of inhabitants at flood risk (1); tourism in non-summer season (1); cultural demands not growing (1)	
Human population-related factors		Harbour density	2	2	1.0	Increasing harbour density (2)	[4,13,15,19,21,22,23,24,25,26,27]
		Human population density	21	14	0.7	Increasing human population density (8); decreasing population density (2); increasing household density (3); no neighbouring communes (1)	
		Other human population-related factors	9	3	0.3	Garbage dump (2); increasing nighttime light (1); increasing urban accessibility	
		Road/rail density	21	13	0.6	Increasing road density (9); increasing distance from roads (2); railway station density (2)	
WB management			11	3	0.3		
Hunting-related variables		Number of feeders	2	0	0.0	Na	Na
		Number of hunters	2	0	0.0	Na	
		Number of hunting grounds	2	0	0.0	Na	
WB abundance		WB abundance/density	3	3	1.0	Increasing wild boar abundance (1); increasing wild boar density (2)	[14,28]
WB habitat			58	43	0.7		
Altitude		Altitude	6	6	1.0	Altitude (3) **; altitude < 500 (reference >1500 m) (1); altitude of 100–500 m (reference >1500 m) (1); altitude 1000–1500 m (reference >1500 m) (1)	[4,10,24,25]
Climatic conditions		Precipitation	7	5	0.7	Mean precipitation (5) **	[4,7,10,29]
		Seasonality	2	1	0.5	Season * herd size (1) **	
		Sun	3	2	0.7	Mean diurnal range (1) **; mean solar radiation (1) **	
		Wind	1	1	1.0	Wind speed (1) **	
		Temperature	6	4	0.7	Na	
Fauna		Other wild animals	1	1	1.0	Presence of wild animals in the village (1)	[13,30]
		Wild suids	1	1	1.0	Area occupied by giant forest hog (1)	
Vegetation		Crops	2	2	1.0	Increased presence of attractive crops in outbreak area (2)	[10,12,14,21,31]
		Vegetation index	2	2	1.0	Normalised difference vegetation index (NDVI) (2) **	
		Landcover	1	1	1.0	Land cover (1) **	
		Protected area	5	2	0.4	Proximity of national park (2)	
		Forest	5	3	(0.6)	Forest density in outbreak area (3) **	
Waterbodies		Water bodies	13	12	0.9	Increasing percentage of area covered by water bodies (3); river density ** (1); increased river density (4); wetlands (3); increasing water vapour pressure (1) **	[4,15,21,22,26,27]
WB suitability		Suitable wild boar area	1	0	0.0	Na	Na
Pig farming system			181	98	0.5		
Biosecurity		Access to farm/pens	8	5	0.6	No gate at entry of farm (1); no lock for each pig pen (1); premises not fenced (2); wild birds observed in pig pen area (1)	[11,14,16,30,31,32,33,34]
		Carcasses/waste management	2	2	1.0	Indiscriminate disposal of offal after slaughter procedure (1); refused disposal of offal after slaughter procedure (1)	
		Cleaning and disinfection	1	0	0.0	Na	
		Disease-related measures	3	0	0.0	Na	
		Foot bath/cloths	9	4	0.4	Disinfection barrier partly or dysfunctional (4)	
		Hygiene practises	2	1	0.5	Mechanical transmission of virus by vehicles and personnel possible (1)	
		Outdoor activities personnel	1	1	1.0	Outdoor activities personnel (1)	
		Pest and rodent control	4	3	0.8	No pests/rodent control (3)	
		Pig introduction/quarantine	2	0	0.0	NA	
		Tools/equipment	3	2	0.7	Tools shared with other farms (1); equipment not washed (1)	
		Arthropod management	3	2	0.7	No insect screens (1); presence of biting insects (1)	
		Vehicles	6	5	0.8	Entry of vehicles on farm (2); no disinfection vehicles (3)	
		Visits on the farm	5	4	0.8	Visits of vets or professionals in high-risk period (3); farm gate buyers in HPR (1)	
Farm management		Bedding	2	2	1.0	No straw as bedding (1); unsafe storage of bedding materials (1)	[11,14,15,16,17,18,30,32,34,35,36]
		Feed and water	14	7	0.5	Swill feeding (3); forage of ASF-infected area (1); feed not stored in closed areas (1); feeding freshly cut forage (1); no control of feed and water (1)	
		Herd size	8	4	0.5	Increasing herd size (4)	
		Management of pigs	16	7	0.4	Animals other than pigs in the farm (1); mixed pigs of different ages on the farm (1); no male pigs on the farm (1); free-ranging pigs (2); sick pigs not separated (1); survivor pig(s) kept by farmer (1)	
		Record keeping	1	0	0.0	Na	
		Slaughter	1	0	0.0	Na	
		Treatments	4	1	0.3	Treatments of internal parasites (1)	
Non-compliance		ASF rules	1	1	1.0	Non-compliance with ASF rules (1)	[15,25,37]
		Registration/census	5	2	0.4	Increased no. of non-registered farms (1); increased proportion of outdoor farms without registration (1)	
		Free range, illegal breeding	1	1	1.0	Pigs illegally bred and free ranging (1)	
		Illegal movements	1	0	0.0	Na	
Pig characteristics		Breed	3	0	0.0	Na	Na
Pig population density		Pig population density	40	26	0.6	Increasing pig density (10); increasing backyard farm pig density (6); increasing low biosecurity farm pig density (5); increasing free ranging pig density (3) Increasing number of pigs per municipality (1), Increasing proportion of pigs in census per 100 pigs (1)	[15,20,21,25,31,34,37,38]
Farm density		Farm density	24	12	0.5	Increasing farm density (4); increasing backyard farm density (1); increasing outdoor farm density (1); increasing medium farm density (1); increasing semi-extensive farm density (1); increasing closed farm density (1); increasing number of farms in each municipality (1); increasing family farm density (1); increasing number of open cycle breeding farms (1)	[15,20,21,25,31,34,37,38]
Pig trade		Pig trade	12	6	0.5	New animals purchased in HRP (1); increasing no. of movements for slaughtering during restriction period (1); increasing no. of pigs purchased last year (1); increasing no. of movements for own consumption in the area (1); increasing no. of live pigs moved from ASF regions (1); increasing no. of outgoing movements from area (1)	[15,17,35,37,39]
Arthropods			9	2 (0.2)			
Ticks		In the area	1	1	1	Area with tick risk (1)	[13,32]
		On pigs/in the farm	1	1	1	Engorged ticks seen on pig by farmer (1)	

* Proportion: For each subcategory, the number of significant variables divided by the total numbers of variables studied. ** highest relative importance/relative contribution of variable to model; HRP: high-risk period; Na: not applicable; No: number.

**Table 2:** Variables studied for their possible association with ASF occurrence in wild boars, analysed in 17 published papers.

Category		Subcategory 2	Total Studies Where Variable Category Was Investigated	No. Studies Where Variable Category Was Significant	Proportion *	Significant Variable	References of Significant Variable
	Subcategory 1						
Timing of the study			20	14	0.7		
Year		Year	20	14	0.7	2015 (3); 2016 (2); 2017 (3); 2018 (2); 2019 (3); 2020 (1)	[40,41,42,43,44]
**ASFV infection pressure**			**15**	**10**	**0.7**		
ASFV infection in outbreak area		Distance outbreak to ASF-affected area	12	8	0.7	Decreased distance to infected area/country (7); ASF reported in neighbouring hexagon in previous period (1)	[43,45,46,47]
		Presence of ASF infection in outbreak area	3	2	0.7	ASF presence in domestic pigs in area (2)	
**Wild boar habitat**			**137**	**75**	**0.5**		
Altitude		Altitude	7	5	0.7	Increasing mean altitude (2), elevation grade (3)	[4,44,48]
Climatic conditions		Precipitation	14	9	0.6	Precipitation **(9)	[4,9,46,48]
		Seasonality	13	1	0.1	Season (1)	
		Sun	1	0	0.0	Na	
		Temperature	17	11	0.6	Mean temperature ** (6); annual range of temperature ** (4); isothermality ** (1)	
Vegetation		Bare land	5	0	0.0	Na	[4,8,20,43,44,46,48,49]
		Crops	12	6	0.5	Increased percentage of area covered by complex cultivation patterns (1); fruit trees and berry plantation (1); rice cultivation (1); rain-fed crops (1); agriculture (1); vineyards (1)	
		Various landcover	2	2	1	Various habitats (1)	
		Herbaceous	5	2	0.4	Na	
		Growth	1	1	1	Increased length of vegetation growing period (1)	
		Vegetation index	3	1	0.3	Normalised difference vegetation index (NDVI) (1)	
		Forest	17	12.0	0.7	Increased percentage of area covered by forest (8); coniferous forest (1); mixed forest ** (1); broad-leaved forest ** (1); transitional woodland–shrub ** (1)	
Water bodies		Water bodies	35.0	21.0	0.6	Increased percentage of area covered by water bodies (8); wetlands (7); peat bogs (1); soil moisture (2); inland marshes (1); water courses (1); increased water vapour pressure (1);	[4,8,38,43,44,48]
WB suitability		Suitable wild boar area	5	4	0.8	Increased percentage of suitable wild boar area (4)	[45]
**Socio-economic factors**			**58.0**	**38.0**	**0.7**		
Social factors		Poverty-related factors	7	7	1.0	Increased amount of differentiated waste (1); cultural traditions (1); low employment rates (1); low share of energy produced by renewable sources (1); increased micro-criminality (1); increased number of inhabitants at flood risk (1); increased number of reported thefts (1)	[20]
Human population density		Human footprint index	4	2	0.5	Decreasing human footprint index (2)	[4,8,40,42,43,44,48]
		Human population density	21	13	0.7	Increasing human population density (11); increased percentage of urban areas (2)	
		Human population-related	9	7	0.7	Increased percentage of discontinuous urban areas (1); green urban areas **(1); industrial or commercial units (1); mineral extraction sites ** (1); land use (1); nighttime light ** (1); urban accessibility ** (1)	
		Road/rail density	17	9	0.5	Increased road density (6); distance from road grade 4 (1); increased travel time to major cities—grade 4 (longest time) (1); path presence **(1)	
WB management			**55.0**	**14.0**	**0.3**		
Hunting		Feed and water	3	0	0.0	Na	[38,43]
		Number of days hunted	2	1	0.5	Decreasing number of days hunted in the hunting area per year (1)	
		Number of feeders	1	0	0.0	Na	
		Number of hunters	3	0	0.0	Na	
		Number of hunting dogs	3	1	0.3	Decreasing number of hunting dogs used in the hunting area per year (1)	
		Number of hunting farms	1	1	1.0		
WB abundance		WB abundance/density	16	11	0.7	Increased wild boar abundance (2); increased wild boar density (8); wild boar density * distance to previous ASF cases (1)	[8,40,42,43,46,48,49]
		Wild boar distribution index	3	1	0.3	Wild boar distribution index grade 5 (1)	[48]
WB dispersal		Female	8	0	0.0	Na	Na
		Male	8	0	0.0	Na	
		Yearlings	8	0	0.0	Na	
WB population characteristics		Females	1	0.0	0.0	Na	Na
		Piglets	2	0	0.0	Na	
Pig farming			36.0	19.0	0.5		
Pig population density		Farm density	19.0	10.0	0.5	Increased farm density (1); increased proportion of pig farms (5); increased proportion of small pig farms in the area (4)	[4,40,41,42,43,48]
		Pig population density	17.0	9.0	0.5	Increased pig density (2); increased density of pigs in small holdings/backyard farms (2); increased proportion of pigs (3); increased proportion of pigs in small farms (2)	

* Proportion: For each subcategory, the number of significant variables divided by the total numbers of variables studied; ** variable with high relative importance/relative contribution to model prediction; HRP: high-risk period; Na: not applicable; No.: number.

## Supplementary Materials

The following supporting information can be downloaded at: https://www.mdpi.com/article/10.3390/v17020192/s1, Table S1: Search strings for literature review; Table S2: Statistical models used in the 48 included articles to study variables possibly associated with ASF occurrence. Ref. [51] is cited in the Supplementary Materials.
