# Supplementary material for: A Systematic Literature Review of Variables Associated with the Occurrence of African Swine Fever
Source: Viruses. 2025 Jan 30;17(2):192. doi: 10.3390/v17020192 (PMC11860392; doi:10.3390/v17020192)
Supplement: Supplementary file 1 [file viruses-17-00192-s001.zip › viruses-3402469-supplementary.pdf]

# Supplementary materials

### Table S1: Search strings for literature review

| Database | Search string |
|---|---|
| Web of Science Core collection | (((TS=(pig* OR porcine OR "Sus domesticus" OR "Sus scrofa" OR swine OR "Wild boar*")) AND TS=("African swine fever*" OR ASF OR ((ASFV OR ASFVs) NEAR/5 (disease* OR infection*)))) AND TS=(Occurren* OR persisten* OR spread* OR introduction OR presence OR recurren* OR incursion)) AND TS=(determinant OR determinants OR factor OR factors) AND English (Languages) and Books or Book Chapters (Exclude – Document Types) |
| PubMed | (("pig"[Title/Abstract] OR "pigs"[Title/Abstract] OR "piglet*"[Title/Abstract] OR "porcine"[Title/Abstract] OR "Sus domesticus"[Title/Abstract] OR "Sus scrofa"[Title/Abstract] OR "Swine"[Title/Abstract] OR "Swine"[MeSH Terms] OR "wild boar*"[Title/Abstract]) AND (("african swine fever*"[Title/Abstract] OR "ASF"[Title/Abstract] OR "African Swine Fever"[MeSH Terms] OR ("ASFV"[Title/Abstract] OR "ASFVs"[Title/Abstract] OR "African Swine Fever Virus"[MeSH Terms])) AND ("disease*"[Title/Abstract] OR "infection*"[Title/Abstract] OR "Disease outbreaks"[MeSH Terms] OR "Infections"[MeSH Terms] OR "Swine Diseases"[MeSH Terms:noexp])) AND ("occurren*"[Title/Abstract] OR "persisten*"[Title/Abstract] OR "spread*"[Title/Abstract] OR "recurren*"[Title/Abstract] OR "presence"[Title/Abstract] OR "incursion"[Title/Abstract] OR "introduction"[Title/Abstract]) AND ("Determinant"[Title/Abstract] OR "determinants"[Title/Abstract] OR "factor"[Title/Abstract] OR "factors"[Title/Abstract] OR "Protective Factors"[MeSH Terms] OR "Risk Factors"[MeSH Terms])) NOT (review[Publication Type]) |
| Scopus | TITLE-ABS-KEY (determinant OR determinants OR factor OR factors) AND TITLE-ABS-KEY (Occurren* OR persisten* OR spread* OR introduction OR presence OR recurren* OR incursion) AND TITLE-ABS-KEY ("African swine fever*" OR ASF OR ((ASFV OR ASFVs) W/5 (disease* OR infection*))) AND TITLE-ABS-KEY =(pig* OR porcine OR "Sus domesticus" OR "Sus scrofa" OR swine OR "Wild boar*") |
| Cab Abstracts | (((TS=(pig* OR porcine OR "Sus domesticus" OR "Sus scrofa" OR swine OR "Wild boar*")) AND TS=("African swine fever*" OR ASF OR ((ASFV OR ASFVs) NEAR/5 (disease* OR infection*)))) AND TS=(Occurren* OR persisten* OR spread* OR introduction OR presence OR recurren* OR incursion)) AND TS=(determinant OR determinants OR factor OR factors) AND English (Languages) and Books or Book Chapters (Exclude – Document Types) |

**Table S2: Statistical models used in the 48 included articles to study variables possibly associated with ASF occurrence.**

| Model | Number of articles | Reference |
|---|---|---|
| **Bayesian multivariable logistic regression mixed model** | 1 | Martinez-Lopez et al., 2015 |
| **Besag, York and Mollie model** | 3 | EFSA, 2018,<br>EFSA, 2020, EFSA, 2021 |
| **Boosted regression tree model** | 1 | Jiang et al., 2022 |
| **Classification tree for binary data** | 1 | EFSA, 2017 |
| **Cox proportional hazard random-effect model** | 1 | Nurmoja et al., 2020 |
| **Generalized additive model for binary data** | 1 | Podgórski et al., 2020 |
| **Generalized additive model for count data (Poisson distribution)** | 1 | EFSA, 2017 |
| **Generalized Linear Logistic Regression** | 2 | Glazunova et al., 2021; Ito et al., 2022 |
| **Generalized linear mixed model for binary data** | 5 | EFSA, 2021 [43]; Huang et al., 2017; Mur et al., 2018, Podgórski et al., 2020, Podgorski et al., 2022 |
| **Hierarchical Bayesian spatio-temporal model for binary data** | 1 | Nurmoja et al., 2020 |
| **Linear regression model** | 3 | Gulenkin et al., 2011; Primatika et al., 2022b |
| **MaxEnt model** | 4 | Ma et al., 2020, Allepuz et al., 2022; Tiwari et al., 2022; Li et al., 2022 |
| **Multiple correspondence analysis** | 1 | Viltrop et al., 2021 |
| **Multivariable conditional logistic regression** | 3 | Fasina et al., 2012; Ramachanderen et al., 2024, Malakauskas et al., 2022 |
| **Multivariable logistic regression** | 7 | Awosanya et al., 2015; Bisimwa et al., 2021; Boklund et al., 2020; Cappai et al., 2018; Chambaro et al., 2020; Dione et al., 2017; Mur et al., 2018; Zakharova et al., 2023 |
| **Negative binomial regression model** | 4 | Cappai et al., 2018; Jurado et al., 2018; Loi et al., 2019; Lee et al., 2022 |
| **Stochastic grid-based compartment model** | 1 | Han et al., 2021 |
| **Zero-inflated Poisson regression model** | 3 | Lim et al., 2021; Vergne et al., 2016 |

Primatika, R.A.; Sumiarto, B.; Widiasih, D.A.; Drastini, Y.; Susetya, H.; Nugroho, W.S.; Putri, M.K. Risk Map of African Swine Fever in Dairi District, North Sumatra Province. In *IOP Conference Series: Earth and Environmental Science*; IOP Publishing: Bristol, UK, **2023**; p. 012013.
